# The activation of retinal HCA2 receptors by systemic beta-hydroxybutyrate inhibits diabetic retinal damage through reduction of endoplasmic reticulum stress and the NLRP3 inflammasome

**DOI:** 10.1371/journal.pone.0211005

**Published:** 2019-01-18

**Authors:** Maria Consiglia Trotta, Rosa Maisto, Francesca Guida, Serena Boccella, Livio Luongo, Cornel Balta, Giovanbattista D’Amico, Hildegard Herman, Anca Hermenean, Claudio Bucolo, Michele D’Amico

**Affiliations:** 1 Department of Experimental Medicine, University of Campania “Luigi Vanvitelli”, Naples, Italy; 2 Institute of Life Sciences, Vasile Goldis Western University of Arad, Arad, Romania; 3 Department of Biochemistry and Molecular Biology, University of Bucharest, Bucharest, Romania; 4 Department of Biomedical and Biotechnological Sciences, School of Medicine, University of Catania, Catania, Italy; 5 Center for Research in Ocular Pharmacology—CERFO University of Catania, Catania, Italy; University of Roehampton - Whitelands College, UNITED KINGDOM

## Abstract

**Objective:**

The role of the hydroxycarboxylic acid receptor 2 (HCA_2_) in the retinal damage induced by diabetes has never been explored. In this context, the present study highlights an upregulation of retinal HCA_2_ receptors in diabetic C57BL6J mice. Moreover, we illustrate that HCA_2_ receptors exert an anti-inflammatory effect on the retinal damage induced by diabetes when activated by the endogenous ligand β-hydroxybutyrate,

**Methodology:**

Seven-to-10-week-old C57BL6J mice were rendered diabetic by a single intraperitoneal injection of streptozotocin (75 mg/kg of body weight) and monitored intermittently over a 10-week period extending from the initial diabetes assessment. Mice with a fasting blood glucose level higher than 250 mg/dl for 2 consecutive weeks after streptozotocin injection were treated twice a week with intraperitoneal injections of 25-50-100 mg/kg β-hydroxybutyrate.

**Results:**

Interestingly, while the retinal endoplasmic reticulum stress markers (pPERK, pIRE1, ATF-6α) were elevated in diabetic C57BL6J mice, their levels were significantly reduced by the systemic intraperitoneal treatment with 50 mg/kg and 100 mg/kg β-hydroxybutyrate. These mice also exhibited high NLRP3 inflammasome activity and proinflammatory cytokine levels. In fact, the elevated levels of retinal NLRP3 inflammasome activation markers (NLRP3, ASC, caspase-1) and of the relative proinflammatory cytokines (IL-1β, IL-18) were significantly reduced by 50 mg/kg and 100 mg/kg β-hydroxybutyrate treatment. These doses also reduced the high apoptotic cell number exhibited by the diabetic mice in the retinal outer nuclear layer (ONL) and increased the ONL low connexin 43 expression, leading to an improvement in retinal permeability and homeostasis.

**Conclusions:**

These data suggest that the systemic treatment of diabetic C57BL6J mice with BHB activates retinal HCA_2_ and inhibits local damage.

## Introduction

Retinal damage is the most common complication of diabetes and is a major cause of several visual impairments leading to adult blindness [1]. Diabetes-induced retinal damage is linked to interrelated pathways and mediators underlying a chronic low-grade inflammatory state [2, 3]. This results in the increased permeability of the blood-retinal barrier, leading to an ischemic event that drives angiogenesis into the retina.

Recently, intact retina has been reported to express the hydroxycarboxylic acid receptor 2 (HCA_2_) [4, 5], a GiPCR receptor activated by β-hydroxybutyrate (BHB), an endogenous ketone body produced by the oxidation of fatty acids in liver mitochondria when carbohydrates are in short supply [6]. While it is well known that HCA_2_ is predominantly involved in the mediation of anti-lipolytic effects on adipocytes, it also exhibits anti-inflammatory and anti-oxidative properties on immune and epithelial cells [7–16]. Therefore, since retinal HCA_2_ expression has been proven, a protective role exerted by this receptor against diabetic retinal damage could be hypothesized. However, HCA_2_ seems to not be properly activated in the diabetic retina: Ghambir and colleagues have shown that following streptozotocin (STZ) administration, diabetic C57BL6J mice exhibited low endogenous BHB serum levels. These levels were insufficient to significantly activate the HCA_2_ receptor and thereby protected the retina from diabetes-induced damage [5]. This led to the hypothesis of an exogenous supply of BHB in this model to achieve a proper HCA_2_ activation.

Based on the evidence, the present study aims to investigate whether an exogenous supply of BHB by systemic treatment can activate retinal HCA_2_ and inhibit local damage in diabetic C57BL6J mice. Moreover, the study also aims to investigate the involvement of retinal ER stress and NLRP3 inflammasome in the actions of BHB, taking into account that BHB can also be an inhibitor of endoplasmic reticulum (ER) stress and of the NOD-like receptor protein 3 (NLRP3) inflammasome [17–23]. While the activation of ER stress exerts a proapoptotic effect [24–25], the NLRP3 inflammasome heralds the onset of changes in the retina, resulting in the attraction of neutrophilic leukocytes, increased permeability and retinal damage [26]. In summary, in this study, we aimed to investigating the beneficial effect of exogenously supplied BHB on i) apoptotic cells in the retina; ii) ER stress and NLRP3 inflammasome markers; and iii) proinflammatory cytokines IL-1β and IL-18 levels.

## Materials and methods

### Animals and experimental design

Seven to 10-week-old C57BL6J mice, housed in a controlled environment (21–23°C, 12–12 h light-dark cycle and a humidity of 55–60%) and fed on a standard chow pellet diet and water *ad libitum*, were used for a diabetes model. Type-2 diabetes was induced in the mice with a single intraperitoneal (i.p.) injection of streptozotocin (STZ, 75 mg/kg of body weight) (Chem Cruz Biochemicals), freshly dissolved in 10 mM citrate buffer (pH 4.5) [1]. To confirm diabetes development, defined by a fasting blood glucose level higher than 250 mg/dl in 2 consecutive weeks (Glucometer Elite XL; Bayer Corp., Elkhart, IN) from the STZ injection, blood glucose levels were then monitored intermittently over a 10 week period from the initial diabetes assessment, a time frame necessary to the development of retinal alterations leading to diabetic retinopathy [1].

C57BL6J mice (N = 10 per group) were randomized into the following experimental groups: (1) nondiabetic mice (CTRL group), receiving an injection of 10 mM citrate buffer alone; (2) nondiabetic mice receiving twice a week i.p. injection of 25 mg/kg BHB (Sigma, Italy) (CTRL+BHB 25 group); (3) nondiabetic mice receiving twice a week i.p. injection of 50 mg/kg BHB (CTRL+BHB 50 group); (4) nondiabetic mice receiving twice a week i.p. injection of 100 mg/kg BHB (CTRL+BHB 100 group); (5) diabetic mice (STZ group); (6) diabetic mice treated twice a week with i.p. injection of 25 mg/kg BHB (STZ+BHB 25 group); (7) diabetic mice treated twice a week with i.p. injection of 50 mg/kg BHB (STZ+BHB 50 group); (8) diabetic mice treated twice a week with i.p. injection of 100 mg/kg BHB (STZ+BHB 100 group) [27]. All the BHB administrations did not modify the blood pH values. At the end of the 10-week period, blood samples were taken from the tail vein (0.2–0.25 ml) into a heparinized Eppendorf vial [28]. A fraction of the whole blood was used for at room temperature for 30 minutes, was centrifuged at 3000 g for 10 minutes at 4°C to obtain serum, which was collected as supernatant fractions [29]. BHB serum levels were checked at the end of 10 weeks by using the β-Hydroxybutyrate ELISA Kit (ABIN773249 Biocompare, Italy), according the manufacturer’s protocol. The mice were then sacrificed, and the retina was dissected as previously described [1]. Retinal samples were then prepared for the Tunel assay, immunofluorescence or biochemical analysis. This study was carried out according to the recommendations of the National Sanitary Veterinary and Food Safety Authority of Romania. The experimental protocol was approved by the Institutional Ethical Committee for Research of the “Vasile Goldis” Western University of Arad (number 29/17.05.2017), and all efforts were made to minimize animal suffering and the number of animals used.

### In situ detection of DNA fragmentation

TdT-FragEL DNA Fragmentation Detection Kit (Calbiochem, EMD Chemicals, INC, San Diego, USA) was used to assess *in situ* nuclear DNA fragmentation based on terminal deoxy-nucleotidyl transferase (TdT), according to the manufacturer’s protocol. The insertion of biotinylated nucleotides allows chromosomal DNA fragmentation to be visualized with streptavidin-horseradish peroxidase (HRP), and stained with diaminobenzidine (DAB), which generates an insoluble dark brown substrate at the site of DNA fragmentation. Nuclear counterstaining was performed with methyl green solution, included in the kit. Sections were viewed by light microscopy (Olympus BX43, Japan) and digital pictures were analyzed with ImageJ software version 1.4. The apoptotic index was expressed as the percentage of TUNEL+ nuclei *versus* the total number of nuclei previously counterstained with methyl green.

### Immunofluorescence

The paraffin embedded retina sections were deparaffinized and rehydrated in an alcohol gradient (100%, 96% and 70% volume). The sections were washed, and antigen unmasking was performed with sodium citrate buffer (pH 6.0). Slides were blocked with 1% bovine serum albumin (BSA) and 5% normal goat serum in phosphate buffered saline (PBS) solution, washed with PBS and incubated with primary antibody, connexin 43 (sc-59949 Santa Cruz, US), for 2 h at 1:200 dilution. The slides were washed and then incubated with the corresponding secondary antibodies diluted 1:500 (Alexa Fluor dye conjugated) in the appropriate blocking solution for 1 h at room temperature in the dark. Counterstaining of nuclei was performed with DAPI. Stained slides were mounted in fluorescence mounting medium (Sigma Chemical, Italy) and analyzed under a Leica TCS SP8 confocal microscope. The intensity of the connexin 43 fluorescence was analyzed with ImageJ64 software (NIH, Bethesda, Maryland, USA). Five fields were selected randomly from each retina section. These values are presented as percentage fluorescence compared to the control group, which is set at 100%.

### Protein quantitation of retinal samples

Following removal of the retinal samples from the C57BL6J mice, they were immediately frozen in liquid nitrogen and stored at -80°C for the subsequent protein collection. Tissue homogenization was performed in RIPA buffer (R0278 Sigma-Aldrich, USA) supplemented with a protease inhibitor cocktail (11873580001 Roche, USA). Then, to remove any nucleic acid contaminants, tissue lysates were centrifuged at 12,000 rpm for 10 min at 4°C, and the supernatants were collected [30]. The protein content of the supernates, used for ELISA and Western Blotting assays, was determined according the Bio-Rad protein assay (500–0006 Bio-Rad Laboratories, Italy).

### Western blotting assay

Proteins were separated on a 12% PAGE separation gel and then electrotransferred onto a PVDF membrane (IPFL10100 Merck Millipore, Italy). The membrane was blocked for 1 hour at room-temperature with 5% nonfat dry milk and then incubated overnight with the following primary specific antibodies: anti-actin (sc-8432 Santa Cruz, USA), anti-HCA_2_ (A02511 BosterBio, USA), anti-eukaryotic translation initiation factor 2-α kinase (PERK) (sc-377400Santa Cruz, USA), anti-phospho-PERK (pPERK) (sc-32557 Santa Cruz, USA), anti-inositol requiring enzyme 1 (IRE1) (ab37073abcam, Italy) and anti-phospho-IRE1 (pIRE1) (PA1-16927 ThermoFisher, Italy). Horseradish peroxidase (HRP)-conjugated goat anti-rabbit (1:5000 ADI-SAB-300) and anti-mouse (1:5000 ADISAB-110 Enzo, Italy) were used as secondary antibodies and incubated for 1 hour at room temperature. The chemiluminescent signals were revealed and expressed as Densitometric Units (D.U.).

### ELISA tests

Retinal BHB levels were determined using the β-Hydroxybutyrate ELISA Kit (ABIN773249 Biocompare, Italy), following the manufacturer’s instructions. Retinal membrane-anchored transcription factor 6α (ATF-6α) (LS-F9075 LifeSpan BioSciences, Italy), NLRP3 (MBS920134 MyBioSource, USA), apoptosis-associated speck-like protein containing a caspase-1 recruitment domain (ASC) (MBS733407 MyBioSource, USA), caspase-1 (MBS092313 MyBioSource, USA), Interleukin 1β (IL-1β) (SMLB00C R&D System, USA) and Interleukin-18 (IL-18) (ab216165 Abcam, Italy) levels were detected using specific ELISA assays, according to the manufacturer’s protocols.

### Statistical analysis

All values from the in vivo experiments are expressed as the mean ± standard error of the mean (S.E.M.) of N = 10 mice. Statistical significance was assessed by Student’s t-test comparing two experimental groups or one-way analyses of variance (ANOVA), followed by Dunnett’s post hoc test (more than two experimental groups). To reject the null hypothesis, a P-value less than 0.05 was considered significant.

## Results

### Retinal HCA_2_ receptor protein expression and BHB levels in diabetic mice

**Fig 1:**
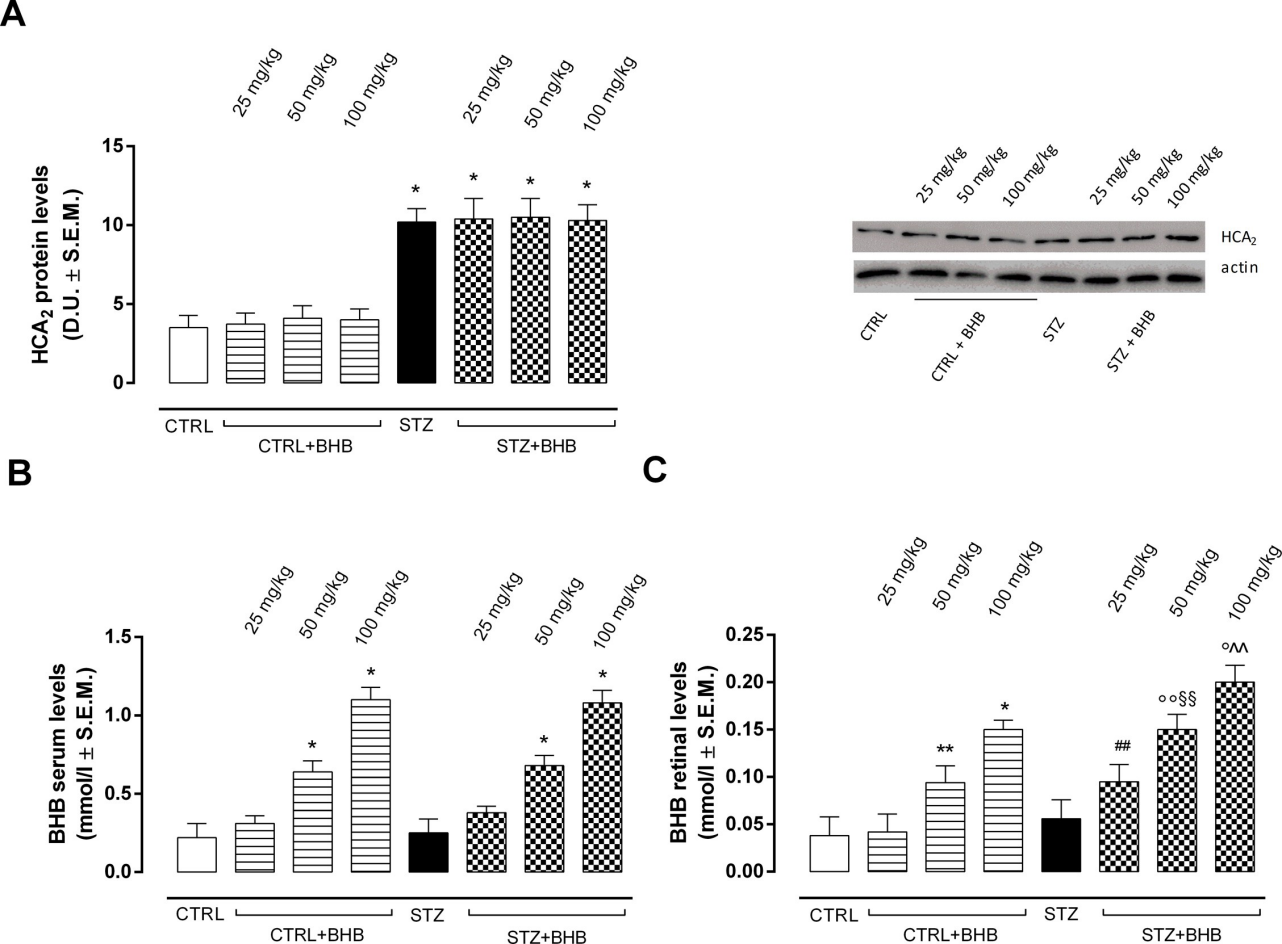
Retinal HCA_2_ receptor protein and BHB levels in serum and retina. **(A)** Detection of retinal HCA_2_ receptor protein levels by Western blot assay; **(B)** Determination of serum BHB levels and **(C)** retina BHB levels by ELISA. Values are expressed as the mean of 10 observations ± S.E.M. CTRL = nondiabetic mice; STZ = diabetic mice; STZ+BHB = diabetic mice receiving BHB. D.U. = Densitometric Units; *P < 0.01 *vs* CTRL; °°P < 0.05 *vs* STZ; °P < 0.01 *vs* STZ; ^##^ P < 0.05 *vs* CTRL+BHB 25; ^§§^ P < 0.05 *vs* CTRL+BHB 50; ^^ P < 0.05 *vs* CTRL+BHB 100.

Western blot analysis showed that STZ mice exhibited significantly increased HCA_2_ receptor protein expression (P < 0.01 *vs* CTRL) compared to CTRL group (Fig 1A). Elevated HCA_2_ receptor protein expression was also detectable in diabetic mice treated with BHB 25-50-100 mg/kg (P < 0.01 *vs* CTRL) (Fig 1A). As expected, STZ mice had serum BHB levels similar to those of CTRL mice, while mice receiving 50 mg/kg and 100 mg/kg BHB both in CTRL and STZ groups exhibited a significant increase in BHB serum levels (P < 0.01 *vs* CTRL) (Fig 1B). Moreover, STZ mice showed retinal BHB levels similar to CTRL group (Fig 1C). These were significantly increased in nondiabetic mice receiving 50 mg/kg (P < 0.05 *vs* CTRL) and 100 mg/kg BHB (P < 0.01 *vs* CTRL), as well as in STZ mice administered with 50 mg/kg and 100 mg/kg BHB (respectively P < 0.05 and P < 0.01 *vs* STZ) (Fig 1C). In particular, diabetic mice receiving the three different BHB doses showed significantly increased retinal BHB levels compared to the nondiabetic mice receiving the same BHB dose (P < 0.05 *vs* CTRL+BHB at the same dose) (Fig 1C)

### Retinal apoptosis and connexin 43 expression in diabetic mice treated with BHB

**Fig 2:**
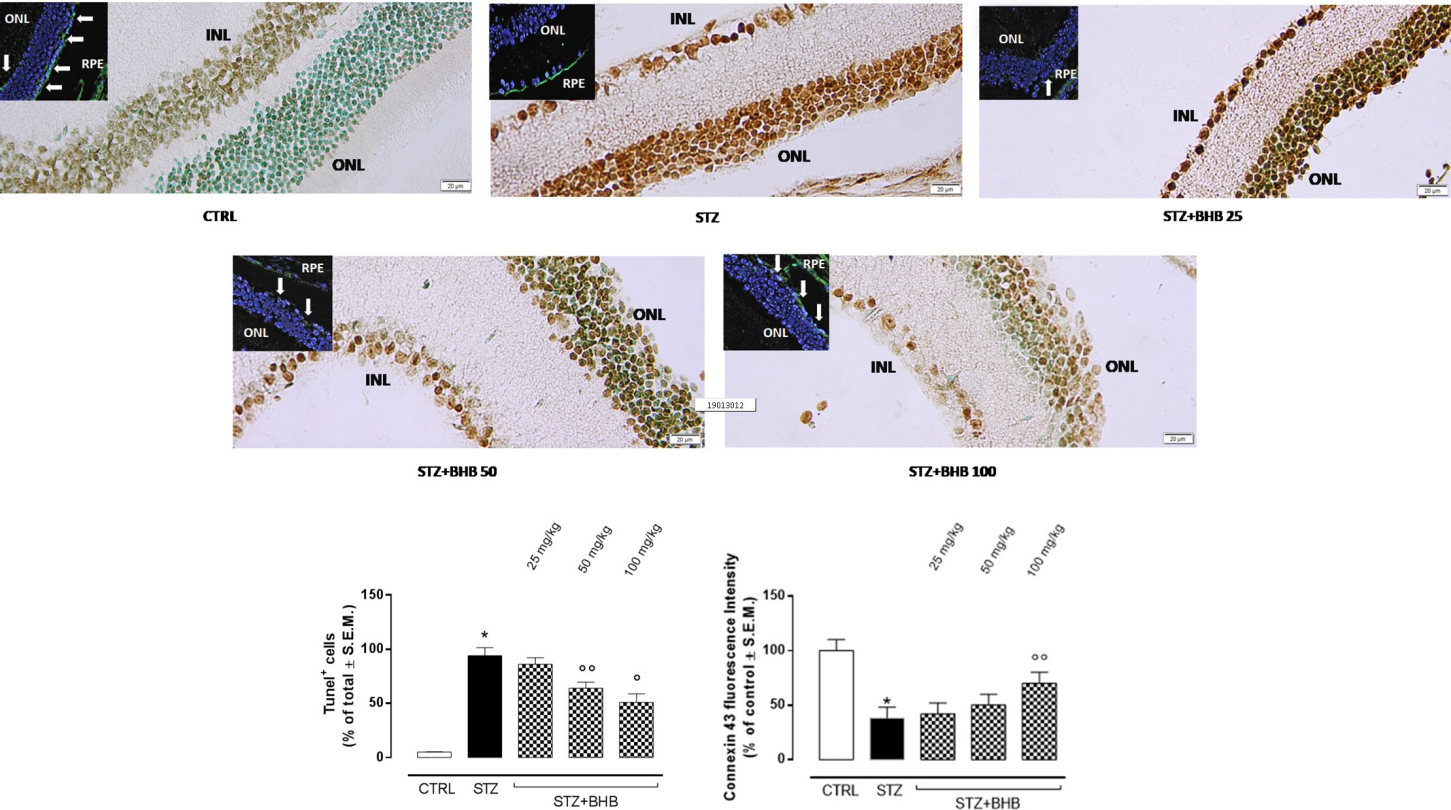
Apoptotic cells and expression of connexin 43 (Cx43) in retinal tissue. Representative Tunel images and connexin 43 immunofluorescence inserts, showing ONL nuclei (in blue) and ONL connexin 43 (in green) labeling. In the immunofluorescence inserts, the green labeling of retinal pigmental epithelium (RPE) was assumed to be a positive control for the connexin 43 staining. Bar graphs represent semiquantification of Tunel^+^ nuclei versus the total number of nuclei previously counterstained with methyl green (mean of the percentage relative to total cells ± S.E.M.) and fluorescence intensity for Cx43 (mean of the percentage relative to control ± S.E.M.) in 5 fields randomly selected from each retina section. INL = inner nuclear layer; ONL = outer nuclear layer; RPE = retinal pigmental epithelium; CTRL = nondiabetic mice; STZ = diabetic mice; STZ+BHB = diabetic mice receiving BHB. *P < 0.01 vs CTRL; °°P < 0.05 *vs* STZ.

The elevation of the expression of HCA_2_ in the retina of diabetic mice was accompanied by a decrease of the expression of the tight junction marker connexin 43 with respect to the nondiabetic mice (P < 0.01 *vs* CTRL). This was accompanied by an increase in apoptotic cell number compared to the control group (P < 0.01 *vs* CTRL). Administration of 50 mg/kg and 100 mg/kg BHB in diabetic mice, thereby activating local HCA_2_, dose-dependently increased the content of connexin 43 in the outer nuclear layer (ONL) (P < 0.01 *vs* STZ) and reduced apoptotic cells (respectively P < 0.05 and P < 0.01 *vs* STZ), which underlie the decrease in retinal damage (Fig 2).

### Retinal ER stress markers and BHB treatment in diabetic mice

**Fig 3:**
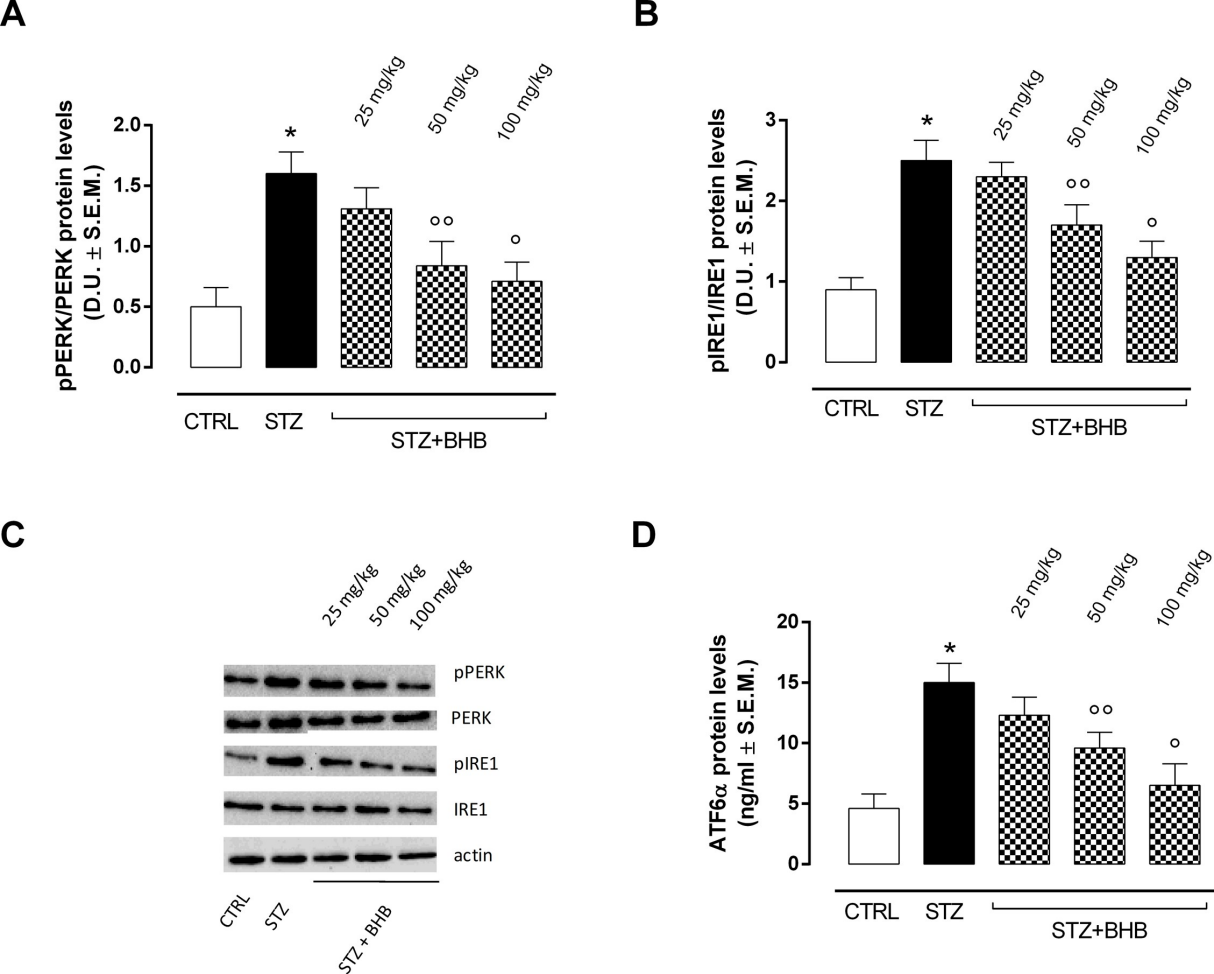
ER stress marker detection. Western blot assays **(A, B, C)** and ELISA **(D)** showing retinal levels of pPERK, pIRE1 and ATF-6α. pPERK and pIRE1 levels were expressed as phosphorylated / total protein ratio. Values are expressed as the mean of 10 observations ± S.E.M. CTRL = nondiabetic mice; STZ = diabetic mice; STZ+BHB = diabetic mice receiving BHB. D.U. = Densitometric Units; *P < 0.01 *vs* CTRL; °°P < 0.05 *vs* STZ; °P <0.01 *vs* STZ.

Data from Western Blot and ELISA assays showed that the retinas of diabetic mice (STZ group) exhibited significantly elevated levels of the ER stress markers pPERK, pIRE1 and ATF-6α compared to the retinas of nondiabetic mice (CTRL group) (P <0.01 *vs* CTRL) (Fig 3). In the retinas of diabetic mice receiving systemic injections of 50 mg/kg and 100 mg/kg BHB (STZ+BHB groups), the ER stress markers were significantly decreased (respectively, P < 0.05 and P < 0.01 *vs* STZ) compared to the STZ group (Fig 3).

### BHB reduces retinal NLRP3 inflammasome activation markers in diabetic mice

**Fig 4:**
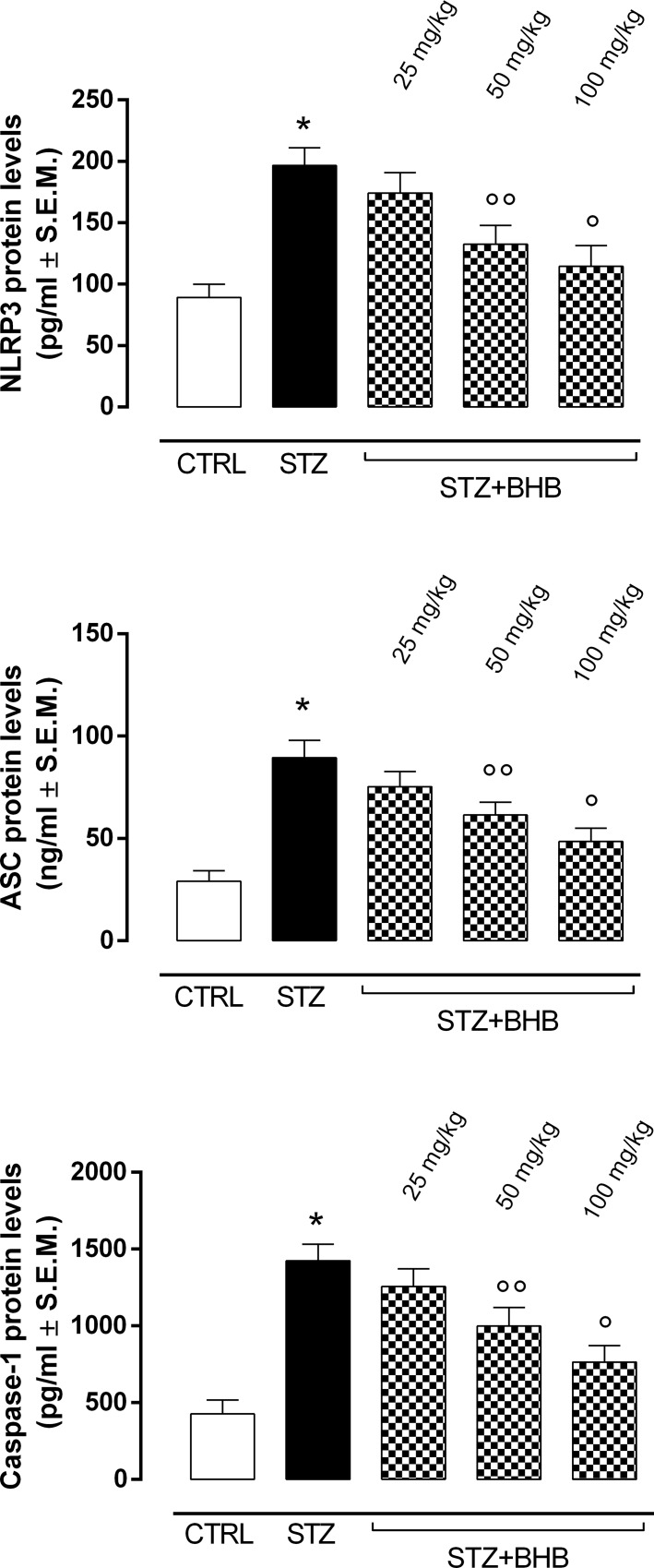
Determination of NLRP3 inflammasome activation markers. Detection of retinal NLRP3, ASC and caspase-1 levels by ELISA. Values are expressed as the mean of 10 observations ± S.E.M. CTRL = nondiabetic mice; STZ = diabetic mice; STZ+BHB = diabetic mice receiving BHB.*P < 0.01 *vs* CTRL; °°P < 0.05 *vs* STZ; °P <0.01 *vs* STZ.

**Fig 5:**
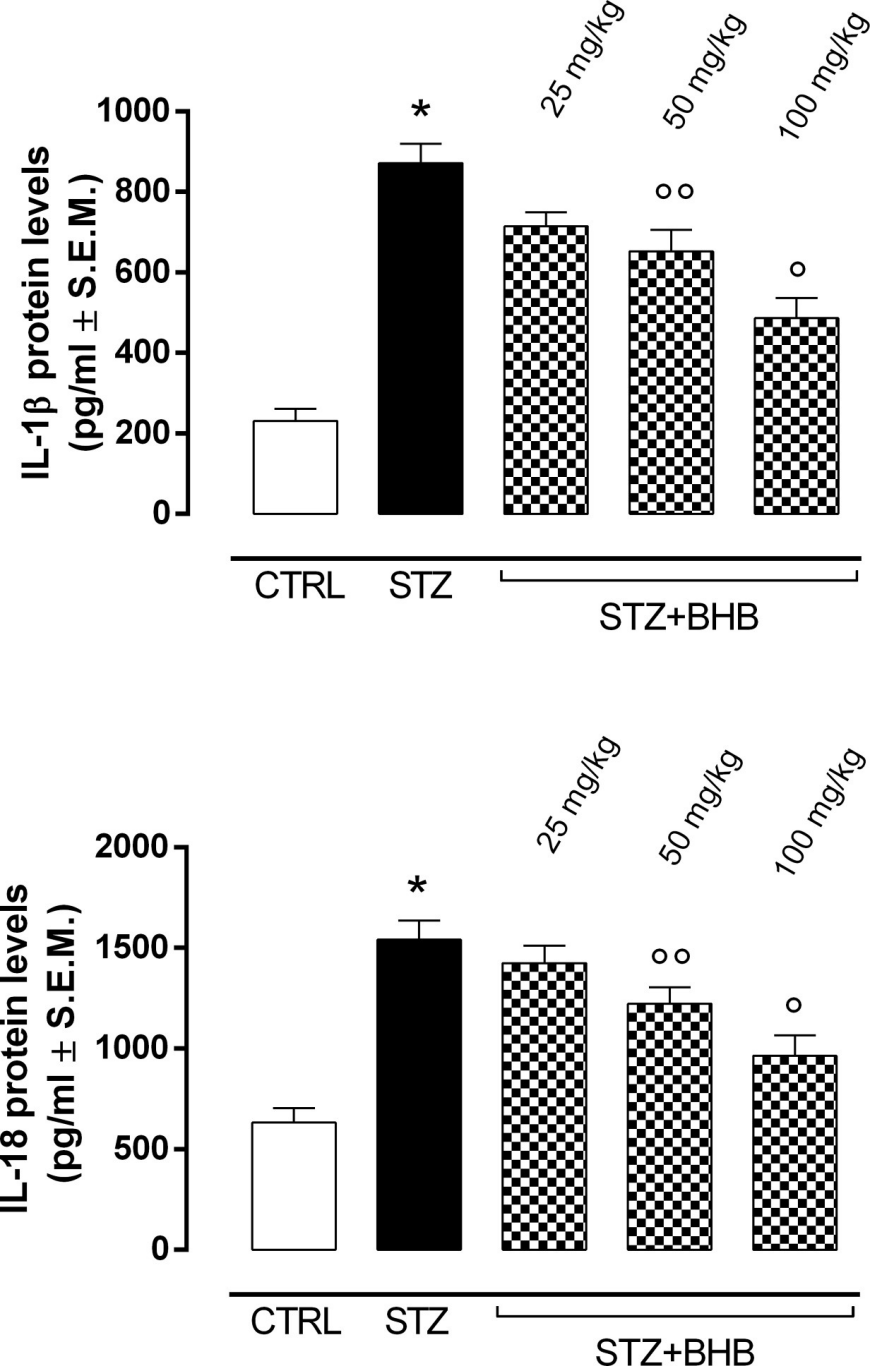
Proinflammatory cytokines IL-1β and IL-18 levels. Determination of IL-1β and IL-18 levels by ELISA. Values are expressed as the mean of 10 observations ± S.E.M. CTRL = nondiabetic mice; STZ = diabetic mice; STZ+BHB = diabetic mice receiving BHB. *P < 0.01 *vs* CTRL; °°P < 0.05 *vs* STZ; °P <0.01 *vs* STZ.

The elevated ER stress markers in diabetic mice (STZ group) were accompanied by significantly increased levels of NLRP3 inflammasome activation markers, such as NLRP3, ASC and caspase-1 (P < 0.01 *vs* CTRL) (Fig 4). In addition, the levels of the proinflammatory cytokines IL-1β and IL-18, which are closely related to NLRP3 inflammasome activity, were significantly increased in STZ mice compared to the nondiabetic group (P < 0.01 *vs* CTRL) (Fig 5). Of note, BHB treatment of the diabetic mice with 50 and 100 mg/kg doses (STZ+BHB group) significantly reduced the NLRP3 inflammasome activation markers previously described (respectively, P < 0.05 and P < 0.01 *vs* STZ) (Fig 4), together with a reduction of IL-1β and IL-18 levels (respectively, P < 0.05 and P < 0.01 *vs* STZ) (Fig 5).

## Discussion

The present study shows that the systemic injection of BHB to STZ-C57Bl6J mice results in retinal protection from diabetic damage through the local activation of the hydroxycarboxylic acid receptor 2, reduction of the ER stress-induced NLRP3 inflammasome activation and a decrease in retinal outer nuclear layer cell death.

HCA_2_ or GPR109A, formerly known as HM74A in humans and PUMA-G (Protein Upregulated in Macrophages by Interferon-γ) in rodents [31], is a G_i_-protein-coupled receptor recognized as an important drug target for treating inflammatory processes [32]. It is endogenously activated by β-hydroxybutyrate (BHB), a ketone body that also exerts an interesting protective effect in different experimental models of neuropathic pain [33]. HCA_2_ receptor is constitutively expressed in all the retinal layers, including retinal pigment epithelial (RPE) cells [4,5,34]. These cells play a critical role in the modulation of the immune and inflammatory responses in retinal physiological conditions, particularly diabetic retinopathy [35–38]. In this context, the present study shows that the HCA_2_ receptor is overexpressed in the retinas of diabetic mice compared to nondiabetic mice. In addition, this HCA_2_ overexpression is accompanied by retinal endogenous BHB levels comparable to that of the nondiabetic mice, data herein described for the first time. In fact, previous studies by Gambhir and colleagues [5] quantified the levels of circulating BHB, without accompanying information on retinal levels. Moreover, the current results reveal that the levels of retinal endogenous BHB in diabetic mice are insufficient to fully activate local overexpressed HCA_2_ receptors, thus expressing the protective effects. Insufficient HCA_2_ stimulation resulted in clear retinal damage in the diabetic animals in our setting. Overall, BHB and HCA_2_ activation are in a very delicate balance, since high BHB levels (>1.8 mmol/L) are largely detrimental and do not optimally activate HCA_2_ receptors [4,39]; conversely, low BHB levels, as in well-controlled diabetes, also do not activate HCA_2_ [40]. Therefore, a strategic increase in BHB bioavailability and proper activation of HCA_2_ might counteract diabetic retinal damage.

As such, we illustrate here that a systemic administration of BHB can increase both serum and retinal BHB levels in nondiabetic and diabetic mice. Particularly, in the latter mice, the retinal BHB enhancement was twice that of the nondiabetic animals. Since we used STZ-C57BL6J mice as a model of diabetic retinal damage without BHB serum elevation [5,41,42], it was assumed that the BHB found in the retina was partially derived from the exogenous BHB through systemic circulation. Although an increase in the retina was expected in the nondiabetic mice, since the ketone body 3-hydroxybutyrate is transported across the blood retinal barrier into the eye [43], the elevation of retinal BHB from exogenous intraperitoneal application represents a novelty in diabetic mice. In the experimental groups, BHB reached significantly higher retinal levels than in the nondiabetic group, likely because it much more easily crossed the retinal blood barrier due to the increased permeability of the damaged barrier in diabetics [44]. This was indicated by a decreased expression of the gap junction integrity marker connexin 43 (Cnx43) in the outer nuclear layer (ONL) of the diabetic retina, paralleled by a higher number of ONL apoptotic cells. Cnx43 plays an important role in maintaining cell survival, since it favors gap junction integrity, thus permitting communication between several cell types, such as endothelial cells, pericytes and retinal Müller cells [45–47]. Of note, the inhibition of retinal Cnx43 expression induced by high glucose levels increases retinal cell apoptosis and derangement in diabetics [1,48,49]. These data are supported by several studies in different experimental settings, showing that reduced Cnx43 expression resulted in increased apoptosis in rat ventricular myocytes [50] and Cnx43-null mice exhibited increased programmed cell death [51]. Interestingly, the diabetic mice treated with BHB exhibited a reduced degree of ONL apoptosis paralleled by a slight restoration of Cnx43 expression, suggesting a protective role of the properly activated HCA_2_ against retinal derangement.

To explain how the activation of HCA_2_ by local BHB exerts retinal protection from the mechanistic point of view, we report that diabetic retinas develop elevated endoplasmic reticulum (ER) stress, manifested here by the increased levels of the ER stress markers phosphorylated proteinkinase (PKR)-like endoplasmic reticulum kinase (pPERK), phosphorylated endoplasmic reticulum to nucleus signaling 1 (pIRE1) and protein ATF-6α [20] in the retina. These proteins are resident transmembrane proteins bound to the ER and are increased following high stress signals to the ER [52] in order to retro-translocate misfolded proteins into the cytoplasm and save the ER from damage through an unfolded protein response [53]. Exogenous BHB reduced the expression levels of all in proteins into the retina, suggesting a reduction in ER stress.

Although a reduction of ER stress through pharmacological activation of HCA_2_ by BHB can save cells and tissue from derangements *per sè* [53], it is our opinion that BHB, through HCA_2_ activation, may also influence other downstream events or factors that produce the cellular damage in the retina of diabetics. BHB also emerged as an NLRP3 inflammasome inhibitor in several experimental settings [17–23] where the ER stress is the initiator. NLRP3 is a multiprotein complex composed of the sensor protein NLRP3, the apoptosis-associated speck-like protein containing a caspase-1 recruitment domain (ASC) and caspase-1 [54–56]. With diabetes, NLRP3 is stimulated by the ER stress and cleaves and stimulates caspase-1, leading to secretion and release of the pro-inflammatory cytokines IL-1β and IL-18 [57]. Altogether, these factors lead to cell and tissue damage. Here, the level of retinal damage in the diabetic mice was paralleled by high levels of NLRP3 inflammasome activation markers, such as NLRP3 protein, ASC and caspase-1, and by elevated levels of IL-1β and IL-18 secreted following NLRP3 inflammasome activation, all of which were reduced by systemic BHB application. These results support several studies showing that NLRP3 inflammasome activation is mediated by ER stress and is negatively involved in retinal damage induced by diabetes [56,58–65]. Although we cannot distinguish between a direct inhibition of NLRP3 and an ER-dependent inhibition by systemic BHB, we can tentatively assume that a positive circuit involving HCA_2_-ER stress and NLRP3 exists in the retina, thereby controlling the diabetic retinal outcome.

**Fig 6:**
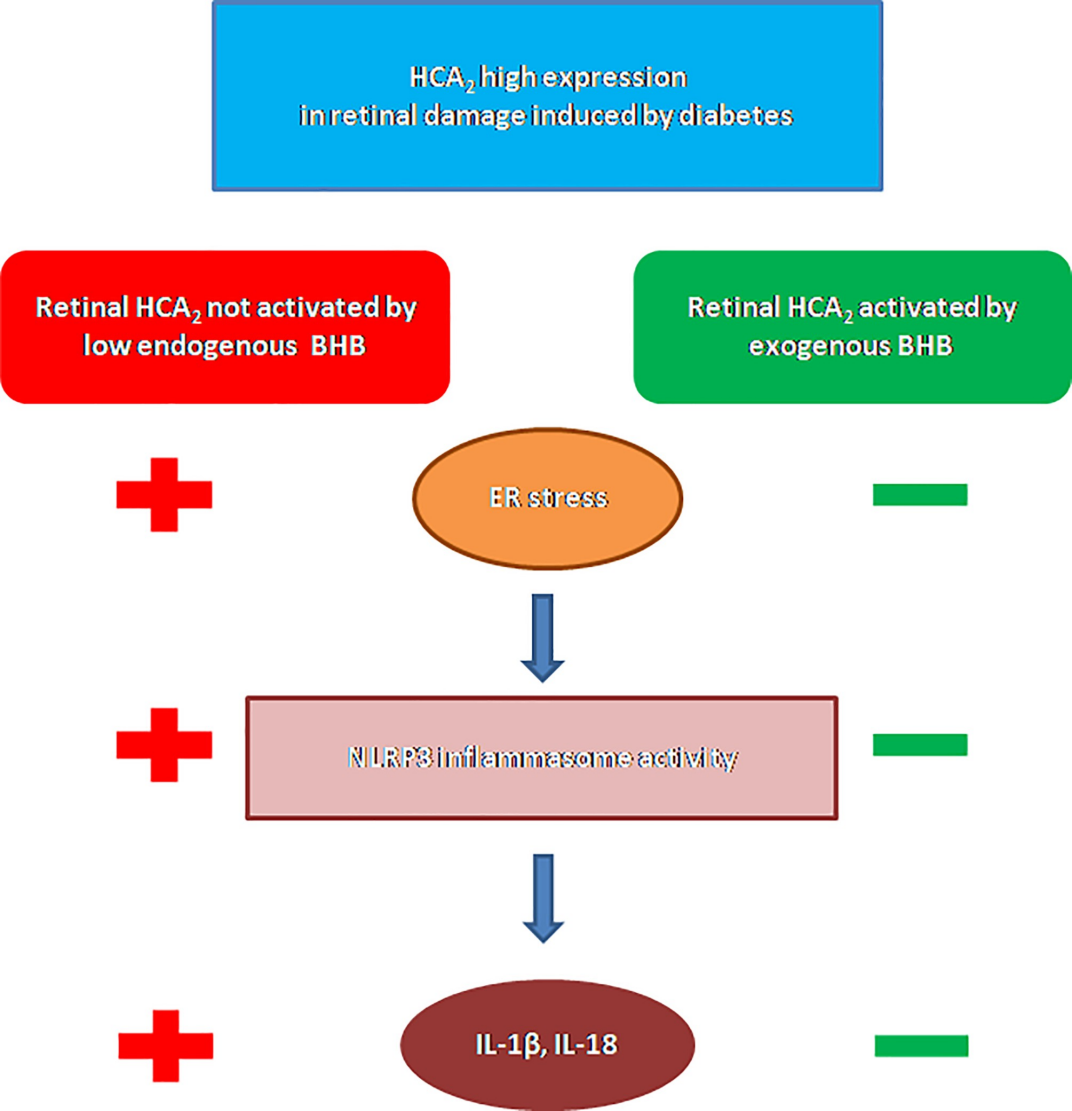
HCA_2_ signaling and activation by BHB in retinal damage induced by diabetes. Although HCA_2_ expression is increased in diabetic retinal damage, the low endogenous BHB levels are not able to activate the HCA_2_ receptors. This results in high levels of ER stress and, consequently, of NLRP3 inflammasome activity, characterized by increased secretion of IL-1β and IL-18. Once BHB is systemic administered, HCA_2_ anti-inflammatory properties are exhibited in the reduction of ER stress, NLRP3 inflammasome activity and proinflammatory cytokines IL-1β and IL-18.

In summary, the present study shows i) overexpression of retinal HCA_2_ with diabetes; ii) increased levels of retinal BHB after systemic administration; iii) reduced ER stress after BHB; iv) reduced NLRP3 inflammasome activity after BHB; and finally, v) reduced retinal damage (Fig 6). These findings pave the way for future investigations on the possible effects of BHB on retinal microglia ramifications, polarization and phagocytosis since BHB has been found to modulate microglia phenotypes associated with the neuro-inflammatory processes underlying retinal degeneration [66]. However, the present data from our experimental setting consistently support the hypothesis that HCA_2_ involvement in the pathogenesis of diabetic retinopathy and its pharmacological activation may offer a new therapeutic target for modulating the inflammatory processes underlying diabetic retinal damage.
